# Supplementary figures and images for: Comprehensive Analysis of the Chitinase Gene Family in Cucumber (Cucumis sativus L.): From Gene Identification and Evolution to Expression in Response to Fusarium oxysporum
Source: Int J Mol Sci. 2019 Oct 25;20(21):5309. doi: 10.3390/ijms20215309 (PMC6861899; doi:10.3390/ijms20215309)

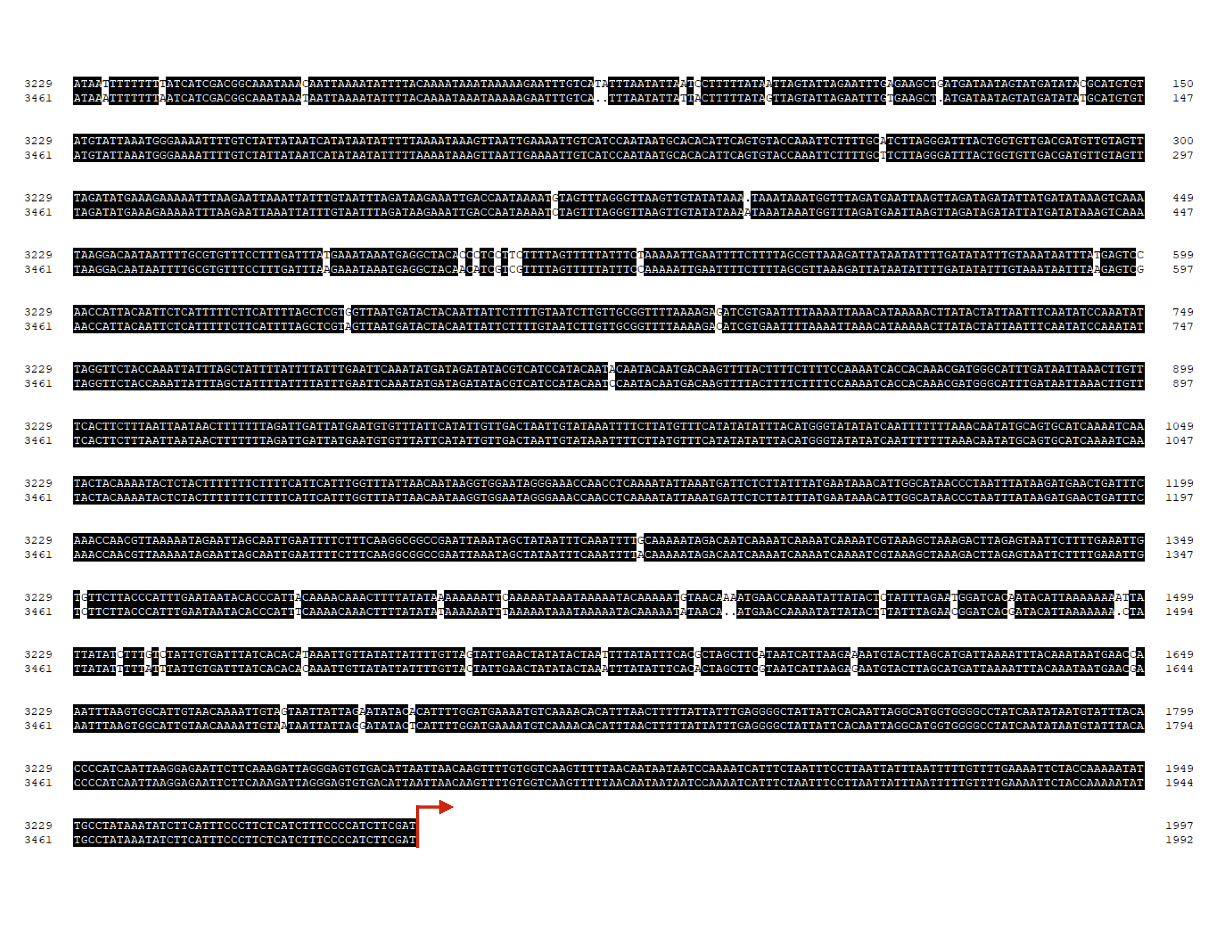

Supplement: Supplementary file 1 [file ijms-20-05309-s001.zip › Figure S2 Genetic variations in the promoter region of CsChi23.tiff]
